# Characterizing the roles of changing population size and selection on the evolution of flux control in metabolic pathways

**DOI:** 10.1186/s12862-017-0962-7

**Published:** 2017-05-25

**Authors:** Alena Orlenko, Peter B. Chi, David A. Liberles

**Affiliations:** 1 Department of Biology and Center for Computational Genetics and Genomics, Temple University, Philadelphia, PA 19122 USA; 2 Department of Molecular Biology, University of Wyoming, Laramie, WY 82071 USA; 3 Department of Mathematics and Computer Science, Ursinus College, Collegeville, PA 19426 USA

**Keywords:** Computational systems biology, Metabolic pathway evolution, Positive directional selection, Fluctuating selection, Fluctuating population size, Co-evolution

## Abstract

**Background:**

Understanding the genotype-phenotype map is fundamental to our understanding of genomes. Genes do not function independently, but rather as part of networks or pathways. In the case of metabolic pathways, flux through the pathway is an important next layer of biological organization up from the individual gene or protein. Flux control in metabolic pathways, reflecting the importance of mutation to individual enzyme genes, may be evolutionarily variable due to the role of mutation-selection-drift balance. The evolutionary stability of rate limiting steps and the patterns of inter-molecular co-evolution were evaluated in a simulated pathway with a system out of equilibrium due to fluctuating selection, population size, or positive directional selection, to contrast with those under stabilizing selection.

**Results:**

Depending upon the underlying population genetic regime, fluctuating population size was found to increase the evolutionary stability of rate limiting steps in some scenarios. This result was linked to patterns of local adaptation of the population. Further, during positive directional selection, as with more complex mutational scenarios, an increase in the observation of inter-molecular co-evolution was observed.

**Conclusions:**

Differences in patterns of evolution when systems are in and out of equilibrium, including during positive directional selection may lead to predictable differences in observed patterns for divergent evolutionary scenarios. In particular, this result might be harnessed to detect differences between compensatory processes and directional processes at the pathway level based upon evolutionary observations in individual proteins. Detecting functional shifts in pathways reflects an important milestone in predicting when changes in genotypes result in changes in phenotypes.

**Electronic supplementary material:**

The online version of this article (doi:10.1186/s12862-017-0962-7) contains supplementary material, which is available to authorized users.

## Background

Understanding the processes that drive lineage-specific evolution is a fundamental challenge in comparative genomics [1]. Many methods have been developed that detect selection, including positive directional selection, at the level of the protein encoding gene. However, proteins function together in pathways and networks, and metabolic pathways are a particularly well understood system. When enzymes under positive selection cluster in a pathway, this can be a sign of directional selection on the pathway, but it can also potentially be explained by negative selection on the pathway with compensatory co-evolution of individual enzymes. These epistatic effects are an important part of the genotype-phenotype map that while ignored by most existing methods, are critical to predicting when changes in genome sequences result in changes to molecular, cellular, and organismal phenotypes. The importance of this work is in characterizing the epistatic nature of the genotype-phenotype map towards this type of prediction of functional shift, using the particular example of metabolic pathways. The same processes that apply to metabolic pathways, apply to other types of pathways and inter-molecular networks, as well as to intra-molecular epistasis [2, 3]. At higher levels of organization, pathways can be redundant and can also have epistatic effects on each other, resulting in ridges in fitness landscapes and more complex patterns of evolution [4]. These complex patterns of evolution are shaped by the interplay of selection on phenotypes, mutational processes, drift, and population genetic processes, which must be understood together to characterize the genotype-phenotype map.

A previous study examined the co-evolution of enzymes in a pathway under negative (stabilizing) selection to preserve pathway flux, which presents a null model for what co-evolution looks like as pathways evolve under more complex scenarios [5]. This previous work, using forward evolutionary simulations [5] and computational analysis of pathways like glycolysis [6] and pyrimidine biosynthesis [7], established the dynamics of the systems, including an important role for mutation-selection-drift balance over longer evolutionary periods. A point to be emphasized is that even when negative selection prevails at the pathway level, individual enzymes can evolve more rapidly than the cumulative function of the pathway as a whole within this paradigm as selection does not act to preserve the activities of an individual enzyme in isolation from the rest of the pathway. If individual enzymes are shifting in their activities relative to the flux through the entire pathway, then their control over the flux of the pathway will shift. This corresponds to potential to shift the flux of the entire pathway through changes to individual enzymes, the potential for mutations of large effect in individual enzyme encoding genes. The stability of flux control on evolutionary timescales can be measured by the number of generations a particular enzymatic step is the slowest and has the most effect on flux.

Before examining selection, one key aspect that has not yet been examined is how the system responds to fluctuations in population size, for example as driven by ecology (e.g. seasonally). An example of this is the seasonal bottlenecking of mammals, reptiles, insects, fungal and plant species [8–11]. Dramatic changes in population size are observed during seasonal and ecological shifts. One hypothesis here is that flux-control stability may be affected by shifting population size. It is hypothesized by us that flux-control step stability may be prolonged due to extreme changes in population size. Similar to shifts in population size, one might also expect interesting dynamics with fluctuating selection.

Moreover, adaptive shifts are expected to pull the population out of the mutation-selection balance and facilitate directional changes in fitness component parameters. Many studies have looked for evidence of pathway-level selection by examining pathways where multiple individual genes show co-temporal evidence for lineage-specific positive selection [12–16]. This will lead to candidate hypotheses, but does not explicitly differentiate between compensatory processes and directional processes. Here the role of fluctuating population size and selection as well as an adaptive directional shift in flux were examined to evaluate patterns of co-evolution and of flux control.

## Methods

### Simulated evolution of metabolic pathways

To evaluate the role of population genetic parameters in biochemical pathway evolution, a population of cells with a key metabolic pathway was evolved under different selective and population genetic schemes. Evolution involves proposing mutations in parameters of the system of ordinary differential equations, followed by selection based upon their effects. The key elements of that scheme were described previously [5] and are summarized here. We simulate the evolution of a metabolic pathway with five reversible reactions and one regulatory loop that controls the rate of production of the first step and one mass action reaction to remove the final product from the system. The simplified kinetic model contains features of glycolysis [17] and is shown in S1. This includes the feedback loop (as an approximation to the regulation of glycolysis) and the synthesis of final metabolite F as analogous to pyruvate in a linear pathway. The model is described by a system of ordinary differential equations where reactions are represented by reversible Michaelis-Menten kinetics [18]. Each enzyme has parameters for enzyme concentration [Enzyme] (mmol/l), the catalytic constant (kcat) (mmol/l/s), the Michaelis constant for the substrate (KM) (mmol/l), the reversible catalytic constant (kcatr) (mmol/l/s), and the Michaelis constant for the product (KMr) (mmol/l). The kinetic model has a single inhibitory reaction that is described in the system by the inhibition constant KI (mmol/l). Additional dynamics include a constant influx of metabolite A and a mass action reaction utilizing F. The steady state solution of that system is calculated using the COPASI environment [19]. Below, we describe two evolutionary simulation frameworks with explicit and non-explicit populations of cells containing the pathways.

First, we have introduced a forward evolutionary simulation framework where a population of cells (an explicit population) containing the synthetic pathways evolves with Wright-Fisher dynamics with mutation and weighted sampling with replacement between generations. We can assign different mutation rates and effects and use various selective schemes to evaluate the fixation of introduced mutations in order to test how the synthetic metabolic pathways evolve. Each forward simulation was repeated 5 times. Mutations were introduced with a probability of 1.5*10^−2^ per parameter per individual per generation. The mutational effects on the catalytic rate constant and enzyme concentration (both indicated by p below) are derived from a standard normal distribution with variable mean ${\mu}_{n_1}$,

1 $$ {\mu}_{n_1}={-0.01 e}^{c^{\ast}\bullet {p}_{n_1-1}}. $$

The mutational effects on the binding constants (K) are described by a standard normal distribution with a variable mean ${\mu}_{n_2}$,

2 $$ {\mu}_{n_2}=\frac{1}{{-0.01 e}^{c\bullet {K}_{n_2-1}}} $$

The index value c is used to scale the mutational effects, with the following values for each constant:

3 $$ c=\left\{\begin{array}{c}{2.5}^{\ast }{10}^{-2}, enzyme\  concentration\\ {}{2.5}^{\ast }{10}^{-2}, inhibition\  constant\\ {}{1.0}^{\ast }{10}^{-2}, catalytic\  constant\\ {}3.{3^{\acute{\mkern6mu}}}^{\ast }{10}^{-4}, reversible\  catalytic\  constant\\ {}1, product\  constant\\ {}3.{3^{\acute{\mkern6mu}}}^{\ast }{10}^{-2}, reversable\  product\  constant\end{array}\right. $$

This mutational scheme allows for scaling across orders of magnitude in kinetic parameters and generates a distribution of mutational effects with a bias towards slightly degrading change that is dependent upon the activity and expression level of the protein.

Fitness of an individual is described as

4 $$ {F}_1=\frac{1}{1+{\left({e}^{-\left( flux-650\right)}\right)}^{0.07}} $$

Values in this logistic function control the asymptotic fitness and the gradient of the flux to fitness relationship. As enzymes reach limits of adaptation because of the ability to utilize products, so do pathways, where the end products are also subjected to the rules of binding and catalysis. The asymptotic control of 650 and slope of 0.07 are arbitrary, but are chosen to reflect the ultimate utilizable flux.

Each of these simulations was run until the point of mutation-selection balance was reached. The point of mutation-selection balance was determined by the stability of the fitness of the median individual across generational time as assessed by observation of approximately equal rates of positive and negative changes.

Another evolutionary simulation framework used a scheme where the Kimura fixation probability was used to evaluate the fixation of proposed mutations, eliminating an explicit population and any probability of multiple segregating changes and representing the population by a single wild type individual. We have

5 $$ \psi =\frac{1-{e}^{-2 c{N}_e s p}}{1-{e}^{-2 c{N}_e s}} $$

to represent the fixation probability, where N_e_ is the population size, c is the ploidy (haploid, c = 1), s is the selective coefficient (s = f’/f_0_–1, where f’ is the fitness after mutation and f_0_ before) and p is the initial frequency of the allele in a population. The initial frequency p was set to ½ rather than 1/N for computational efficiency, giving the property that a neutral mutation has a 50% chance of fixation, which scales the selective coefficient. The effects of population size contributed to experimental results in rising from a 0.5 frequency to fixation and the introduction of new mutations was independent of population size.

This experimental setup with the Kimura fixation probability was run for 200,000 generations per experimental replicate and the rate-limiting step length was calculated as was previously described [5]. Each instance of population size was run for 30 replicates. Sensitivity analysis was used to calculate the rate-limiting step for a generation by changing one reaction step at a time by 10%, while others were fixed, and calculating the difference between the original and perturbed state fluxes, generating a sensitivity coefficient. When a reaction is rate-limiting, changing the reaction rate has a larger effect than it does for reactions that are not rate-limiting.

### Experiments with fluctuating population size and fluctuating selective pressure

A subset of experiments used an explicit population. For these experiments with an explicit population, mutations were introduced with a higher rate of 1.5*10^−2^ per parameter per individual per generation (as compared with previous studies). The previously published scheme [5] with selection on flux only was used as the basis for all of the experiments. Simulations with an explicit population had various schemes for fluctuating the population size (summarized in Table 1). Six experiments will be analyzed here: N1 with a periodicity of 360 generations and amplitudes that range between 25 and 225 individuals, N2 with periodicity 45 generations and amplitudes that range between 50 and 150 individuals, N3 with periodicity 360 generations and amplitudes with the range of [50:150] individuals, N4 with periodicity 720 generations and amplitude with the range of [25:225] individuals, N5 with periodicity 45 generations and amplitude with the range of [25:225] individuals, N6 with periodicity 22.5 generations and amplitudes with the range of [25:225] individuals (see Additional file 1: Figure S2 for experimental schemes). Corresponding to each population scheme, three control experiments were examined: the lowest population size, the median population size, and the highest population size. For schemes N2, N3 controls are the experiments with population size 50, 100, 150. For schemes N1, N4, N5 and N6 controls are the experiments with population size 25, 150, and 225.

**Table 1:** A summary of the parameters used across experiments (amplitude and periodicity) is shown

	Amplitude	Periodicity
C_N_25	[25:25]	-
C_N_50	[50:50]	-
C_N_100	[100:100]	-
C_N_150	[150:150]	-
C_N_225	[225:225]	-
N1	[25:225]	360
N2	[50:150]	45
N3	[5-:150]	360
N4	[25:225]	720
N5	[25:225]	45
N6	[25:225]	22.5
C_K_100	[100:100]	-
C_K_10000	[10,000:10,000]	-
C_K_1000000	[1,000,000:1,000,000]	-
K1	[100:1,000,000]	5760
K2	[100:1,000,000]	11,520
K3	[100:1,000,000]	23,040
K4	[100:1000]	23,040
K5	[100:10,000]	23,040
C_0.5	[0.5:0.5]	-
C_1.0	[1.0:1.0]	-
C_1.5	[1.5:1.5]	-
S1	[0.5:1.5]	45
S2	[0.5:1.5]	360

A number of fluctuating population size schemes were implemented in the experiments with a calculated fixation probability for each introduced mutation (Additional file 1: Figures S3A, 3B). The amplitude was set to a range of 100 to 1,000,000 individuals for the following schemes with variable periodicity: K1 5760 generations, K2 11,520 generations, K3 23,040 generations. For the periodicity of 23,040 generations, two more amplitudes were tested: experiment K4 with the amplitude range of 100 to 1000 individuals, and experiment K5 with the amplitude range of 100 to 10,000 individuals. Control experiments correspondingly contain population sizes of 100, 10,000, and 1,000,000.

Two fluctuating selection schemes were tested here by adjusting the asymptote of the fitness parameter that was originally introduced in [5]:

6 $$ {F}_1=\frac{1}{1+{\left({e}^{-\left( flux-{a}^{\ast }650\right)}\right)}^{0.07}} $$

Schemes following the same amplitude range [325, *a* = 0.5; 975, *a* = 1.5] and different periodicity included S1 with periodicity 45 and S2 with periodicity 360. Three controls with *a* set to 0.5, 1.0, and 1.5 were evaluated correspondingly. These changes in the *a* value result in changes to the amplitude.

### Simulations with positive directional selection

In simulating with positive directional selection, for the first 2000 generations of simulations, the asymptotic parameter X was equal to 650 (equilibrium state 1) representing the previously described selection on flux only. The adaptive shift experiment contains the following scheme. Two different asymptotic control parameters X were applied to the fitness function as below.

7 $$ {F}_1=\frac{1}{1+{\left({e}^{-\left( flux- X\right)}\right)}^{0.07}} $$

**Fig. 1:**
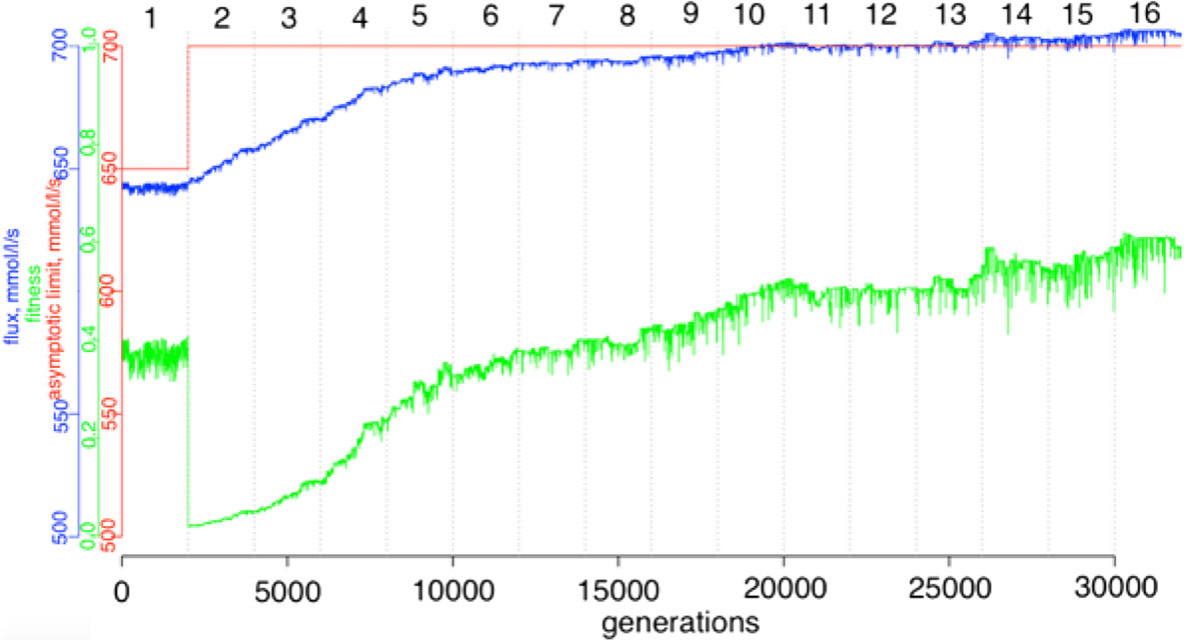
During positive directional selection, fitness (*green*), flux (*blue*) and the selected fitness value (*red*) over the time-course in the experiment with an explicit population are shown

After 2000 generations, X was set to 700, which triggered a fitness recalculation in the system and enabled the adaptation process to begin. After applying a positive selection stimulus (X = 700), a new mutation-selection equilibrium (equilibrium state 2) was established after 28,000 generations (30,000 generations after generation 0) with system adaptation to the new conditions. It was additionally run for 2000 generations after equilibrium state 2 (for a total of 30,000 generations) (Fig. 1).

### Statistical analysis of flux control stability

To assess statistical significance of any step spending a longer period as rate limiting, a permutation test was utilized for the null hypothesis of no flux control stability, which implicitly means that each reaction should have the same average number of consecutive generations that it remains rate limiting. Thus, we chose the average absolute deviation as our statistic of interest and generated its null distribution in each case, in a manner similar to that in [5]. Additionally, bootstrap confidence intervals were constructed by first bootstrapping the replicates, and then bootstrapping the values of consecutive runs within each replicate. Error bars on the corresponding figures indicate 95% confidence bounds, obtained by taking the 2.5th and 97.5th percentiles from the bootstrap sampling distribution.

### Examination of evolution and co-evolution in experiments under positive directional selection

Simulations where positive directional selection was applied using methodology that was similar to that which has been described previously with an explicit population of individuals [5]. Briefly, coevolution was estimated using the kinetic parameter values (k_cat_, k_catr_, K_m_, K_mr_ [enzyme]) in the median individual at each generation for each 2000 generations. Every 2000 generations, complete linkage clustering was performed using absolute correlations as a measure of relatedness between the rates of change of parameters of the system. The largest clusters that were significant at the 0.05 level were used to identify co-evolving parameters. A total of 16 periods of evolution were tested and subjected to statistical analysis.

### Statistical analysis of co-evolution during selection for increased flux

Testing whether the rate of change of flux was associated with the amount of inter-molecular co-evolution was a hypothesis to be tested here. Each block of 2000 consecutive generations was examined, for the change in flux from its beginning to end, and the number of inter-molecular clusters identified (reflecting parameter values from different enzyme steps that showed evidence for co-evolution). A mixed-effects model was employed, to account for the multi-level data structure induced by the fact that observations within each replicate are correlated. Our statistic of interest is the slope main effect describing the extent to which an increase in the rate of change of flux is associated with increased inter-molecular co-evolution. We analyzed this non-parametrically by utilizing a permutation test to generate a null distribution for the slope.

## Results

It has previously been shown that flux control in metabolic pathways is expected to be unstable under mutation-selection-drift balance with simple population genetic and selective schemes [5]. One question raised from the observed results is when flux control stability might emerge. The evolutionary ecology [8–11] and molecular evolution literatures [20] have emphasized an important role of fluctuating environments or population sizes in complex evolutionary dynamics. Fluctuation of both flux asymptotes (selection levels) and population sizes have been evaluated in forward evolutionary simulation frameworks to examine this in parameter-controlled settings.

### Experiments with an explicit population and a fluctuating population size

**Fig. 2:**
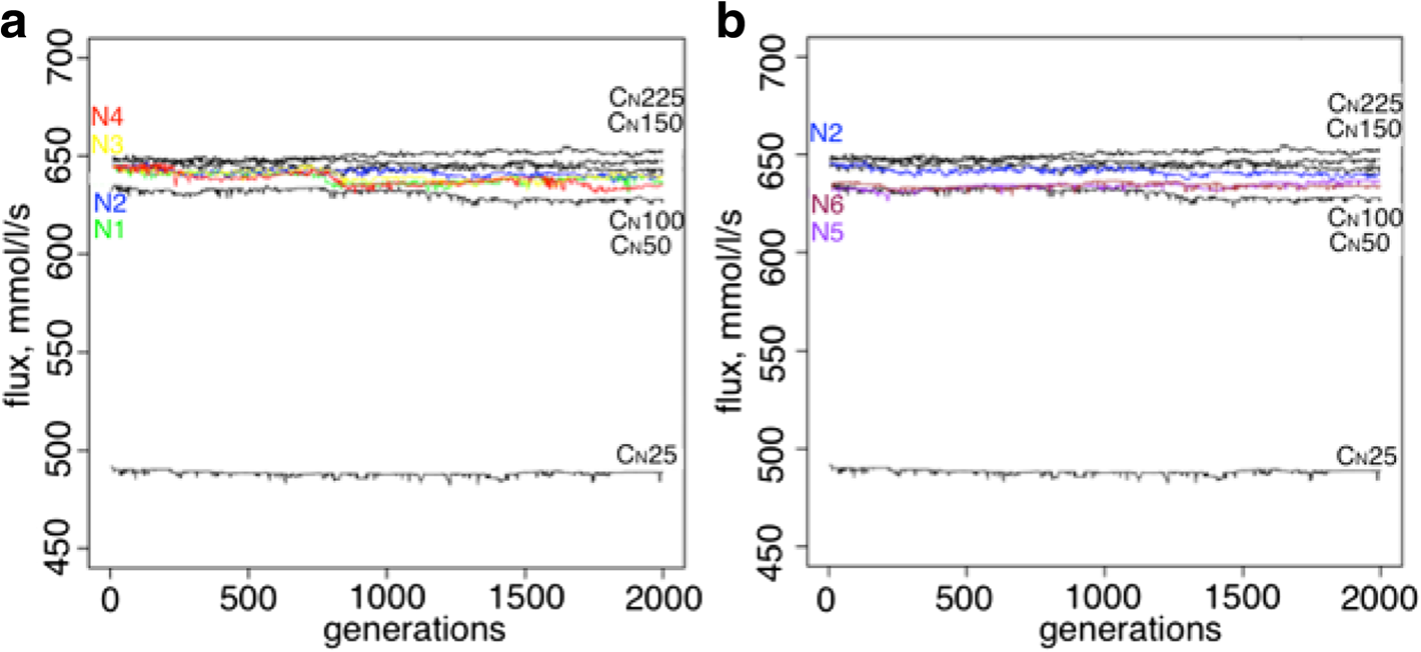
The fluxes from experiments with an explicit population that fluctuated in size are shown. **a**. The fluxes for fluctuating experiments N1 (*green*), N2 (*blue*), N3 (*yellow*), N4 (*red*) are shown. *Black lines* correspond to the control experiments with population sizes 25 (C_N_25), 50 (C_N_50), 100 (C_N_100), 150 (C_N_150), 225 (C_N_225). **b**. The fluxes for fluctuating experiments N2 (*blue*), N5 (*purple*), N6 (*brown*) are shown. *Black lines* correspond to the control experiments with population sizes 25 (C_N_25), 50 (C_N_50), 100 (C_N_100), 150 (C_N_150), 225 (C_N_225)

The analysis started with an explicit population framework that estimates the effect of a fluctuating population size with different amplitudes and frequencies (given a fixed mutation rate and effect distribution) on the evolutionary stability of rate-limiting steps. A number of fluctuating population size schemes and controls with fixed population sizes were tested for stability of rate-limiting steps and are shown here (Table 1, Additional file 1: Figure S2). The schemes (Additional file 1: Figure S2) represent different amplitudes and periodicities of population size fluctuations. Schemes N2 and N5 have the same periodicity, while schemes N1, N4, N5, N6 and N2, N3 have the same amplitude. Rate-limiting step stability was assessed when mutation-selection-drift balance equilibrium was reached and flux changes over the time-course didn’t show any directional fluctuations. Figure 2 shows that fitnesses of all of the experiments do not show local temporal adaptation to fluctuation in the population size, where all observed changes come from compensatory processes under mutation-selection balance with no directional adaptive changes. It is well understood that population size alters the relative roles of drift and selection, with a stronger role for selection in larger population sizes. This is in fact observed in the population size effects in Fig. 2. It is also understood that the dynamics of fluctuating population sizes are driven by the bottlenecks (smaller Ne values).

**Fig. 3:**
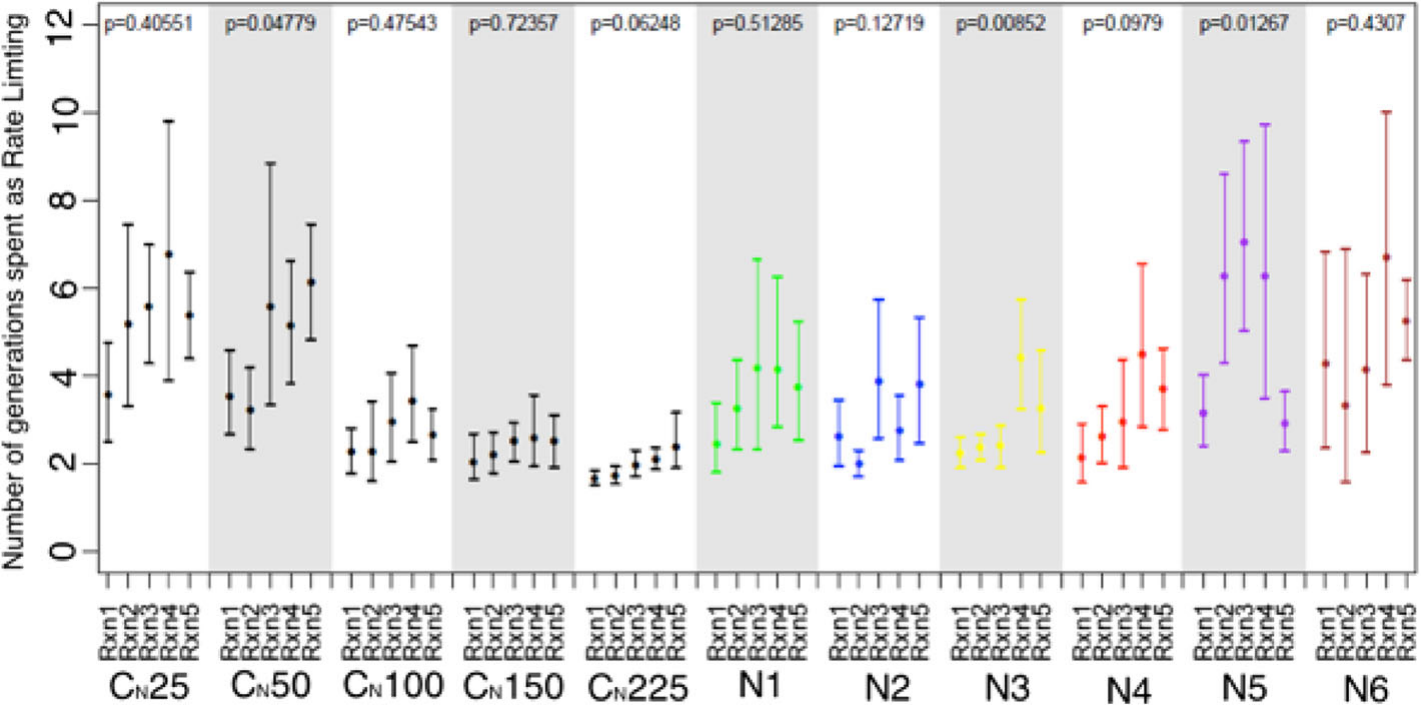
The distributions of the average length of rate-limiting steps between the reactions in the experiments with an explicit population and a fluctuating population size are shown. The schemes for fluctuating experiments N1 (*green*), N2 (*blue*), N3 (*yellow*), N4 (*red*), N5 (*purple*), and N6 (*brown*) are shown. *Black bars* correspond to the control experiments with population sizes 25 (C25), 50 (C50), 100 (C100), 150 (C150), 225 (C225). Nominal *p*-values were obtained via permutation tests with the null hypothesis that the average absolute deviation in the number of consecutive generations for each step was zero

Controls for the experimental schemes were assessed correspondingly at the highest, lowest, and middle point of each scheme: with population sizes of 50, 100 and 150 for schemes N2, N3 and population sizes of 25, 150 and 225 for schemes N1, N4, N5 and N6. Calculation of the average consecutive length of each reaction being rate-limiting (Fig. 3) showed that scheme N5 has a statistically significant difference in the number of consecutive generations spent as rate limiting, across the reactions. This scheme has elevated amplitude as compared to the scheme N2 and N3, but also has a smaller periodicity as compared to schemes N1 and N4 and larger periodicity as compared to scheme N6. Also it could be seen that the experiment with higher amplitude and lower periodicity (N6) has a signal for elevated rate-limiting step stability when compared to N2 and controls C_N_100, C_N_150, and C_N_225, but there is not statistical support for differences across reactions. A further increase in periodicity (N1 and N4) did not result in a signal for elevated rate-limiting step stability. The strongest signal for differences in the consecutive number of rate limiting steps across reactions was observed in N3. However, it should be noted that one of the control experiments, C_N_50, showed a *p*-value for unequal flux control across steps of less than 0.05, at *p* = 0.04779, and this was the control scenario specifically for schemes N2 and N3.

### Experiments with a calculated fixation probability and a fluctuating population size

**Fig. 4:**
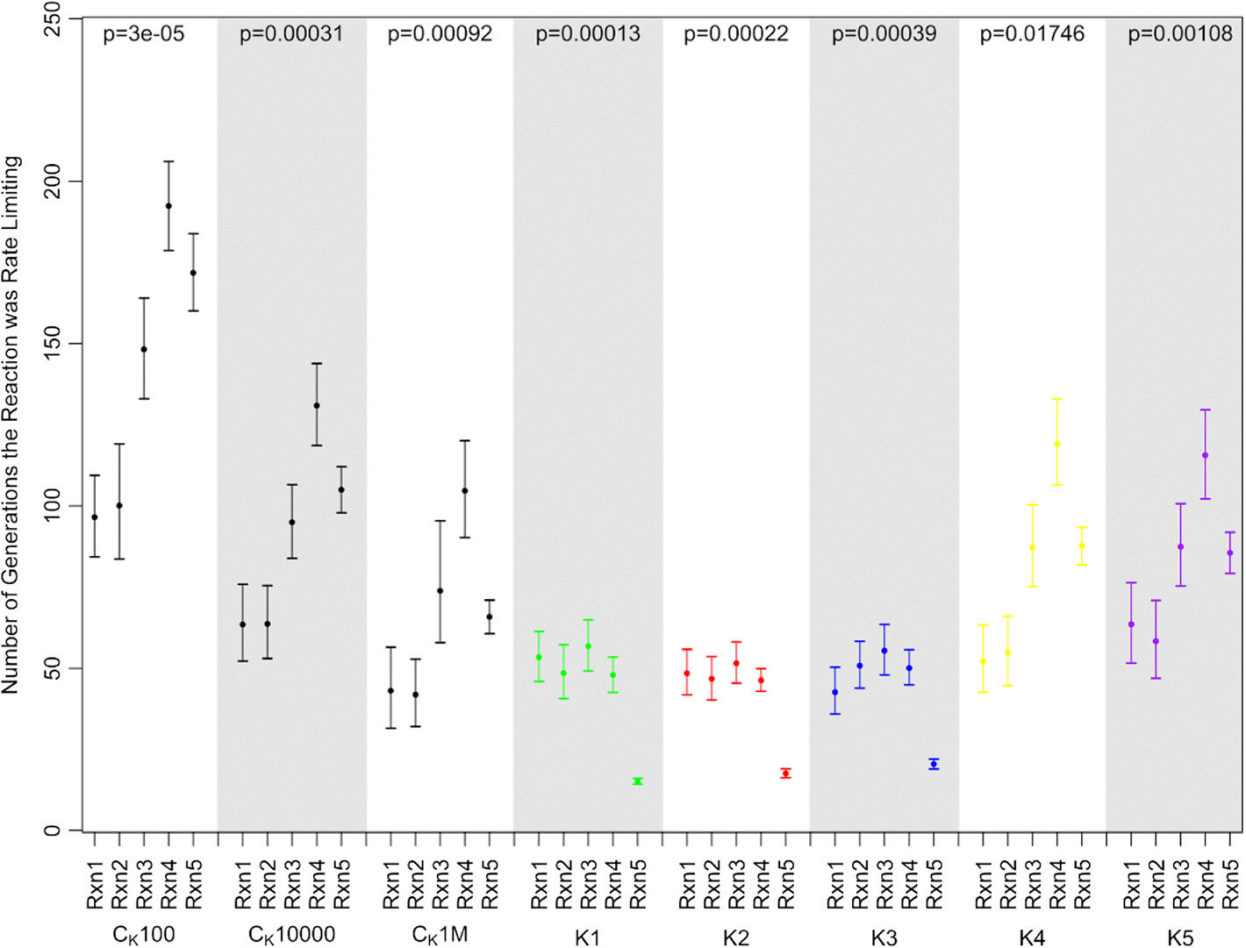
The distributions of the average consecutive length of rate-limiting steps in the experiments with a calculated fixation probability for each mutation and a fluctuating population size are shown. The schemes of fluctuating experiments K1 (*green*), K2 (*red*), K3 (*blue*), K4 (*yellow*), and K5 (*purple*) are shown. *Black bars* correspond to the control experiments with population sizes 100 (C_K_100), 10,000 (C_K_10000), 1,000,000 (C_K_1M).). Nominal *p*-values were obtained via permutation tests with the null hypothesis that the average absolute deviation in the number of consecutive generations for each step was zero

**Fig. 5:**
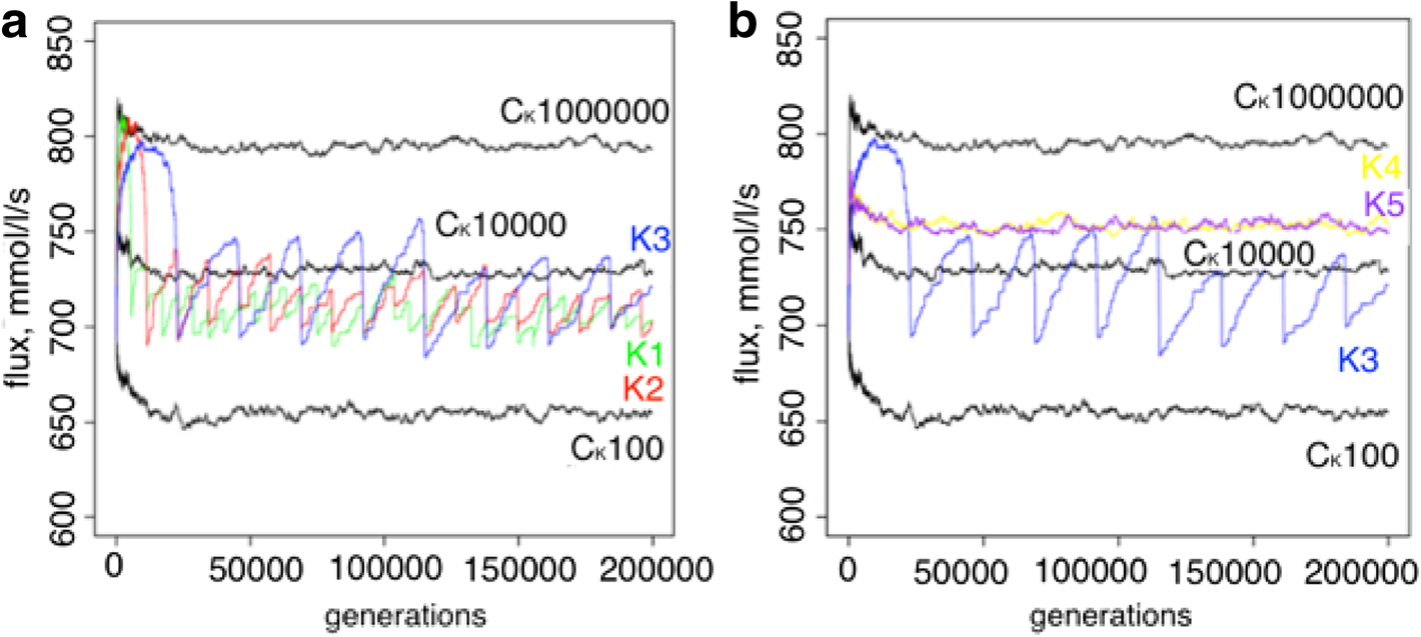
The fluxes of the experiments with a calculated mutational fixation probability and a fluctuating population size are shown. **a**. The fluxes of fluctuating experiments K1 (*green*), K2 (*red*), K3 (*blue*) are shown. *Black lines* correspond to the control experiments with population sizes 100 (C_K_100), 10,000 (C_K_10000), 1,000,000 (C_K_1000000). **b**. The fluxes of fluctuating experiments K3 (*blue*), K4 (*yellow*), K5 (*purple*) are shown. *Black lines* correspond to the control experiments with population sizes 100 (C_K_100), 10,000 (C_K_10000), 1,000,000 (C_K_1000000)

With an explicit population, multiple mutations can simultaneously segregate, an elevated amplitude with an appropriate frequency (tuned to the mutation rate to enable response to environmental changes) in population size fluctuations increased the evolutionary stability of rate-limiting steps. However, because the increase of the amplitude is computationally expensive in the explicit population framework, an alternative population framework involving the Kimura fixation probability that allows the implementation of any population size in a computationally feasible manner was used. Five different schemes with an explicit fixation probability were studied here in order to estimate various ranges of amplitudes. First, an elevated amplitude [100, 1,000,000] was tested on different periodicity schemes (K1, K2, K3) corresponding to consecutive doubled increases in periodicity. The resulting stability increase slightly correlates with the periodicity increase (Fig. 4, Table 2). Two other amplitude schemes K4 [100, 1000] and K5 [100, 10,000] were examined with both having the same periodicity as scheme K3. Scheme K4 showed the largest average rate-limiting step stability out of all tested schemes. As in the previous experimental design, the specific range of both periodicity and amplitude can result in a signal of elevated rate-limiting step stability. Analysis of finesses revealed that schemes K1-K3 represent adaptive behavior of the system (Fig. 5). Here, the range of population size fluctuations independent of the periodicity represent local directional selection on flux. This can be seen in the fluctuations of the flux that follow the fluctuations in population size. This behavior changes when the amplitude of population size fluctuations is reduced to be less extreme causing less intense selective pressure, as can be seen for experiments K4 and K5. It should be noted that the asymmetry in the responses to increasing and decreasing flux reflect the relative frequencies of flux increasing (rarer) and flux decreasing mutations (more common), according to the mutational scheme that has been designed. This reflects the natural bias towards “deleterious” mutations that is known. Controls for the experimental schemes were assessed correspondingly at the highest, lowest, and middle point of scheme K1, K2 and K3: with population size 100, 10,000 and 1,000,000.

**Table 2:** The fraction of time each reaction spent rate-limiting for the experiments with fluctuating population size and two distinct population frameworks, with an explicit population and with an explicit fixation probability are shown

	Reaction1	Reaction2	Reaction3	Reaction4	Reaction5
Explicit population					
C25	0.08	0.13	0.23	0.29	0.27
C50	0.12	0.12	0.21	0.23	0.32
C100	0.11	0.14	0.22	0.32	0.21
C150	0.14	0.15	0.22	0.25	0.24
C225	0.12	0.14	0.21	0.26	0.27
N1	0.10	0.13	0.23	0.28	0.26
N2	0.13	0.10	0.27	0.19	0.31
N3	0.10	0.11	0.16	0.38	0.25
N4	0.07	0.12	0.20	0.31	0.29
N5	0.08	0.20	0.32	0.27	0.12
N6	0.11	0.06	0.20	0.32	0.30
Fixation Probability					
C100	0.04	0.04	0.15	0.30	0.47
C10000	0.04	0.04	0.14	0.29	0.48
C1000000	0.04	0.04	0.16	0.31	0.46
K1	0.17	0.17	0.20	0.25	0.21
K2	0.17	0.16	0.20	0.23	0.24
K3	0.14	0.16	0.19	0.25	0.27
K4	0.03	0.04	0.14	0.32	0.47
K5	0.05	0.04	0.15	0.30	0.47

There is also a noticeable decrease in the evolutionary stability of rate-limiting step at reaction 5 in some of the experiments including controls. Additional tests for differences in rates of mutational acceptance at each step did not show any deviations. Moreover, examination of the fraction of time each reaction was rate-limiting showed that all reactions have somewhat equal time being rate-limiting in all of the experiments (Table 2), although less equal than suggested by the rate limiting step stability. These findings suggest that the reduced evolutionary stability of rate limitation for reaction 5 is connected to the location of reaction as the last step in the pathway and the interplay of the pathway flux with the mass action procedure. Changing the mass action procedure to ensure that it cannot be rate-limiting causes this evolutionary instability to disappear.

**Fig. 6:**
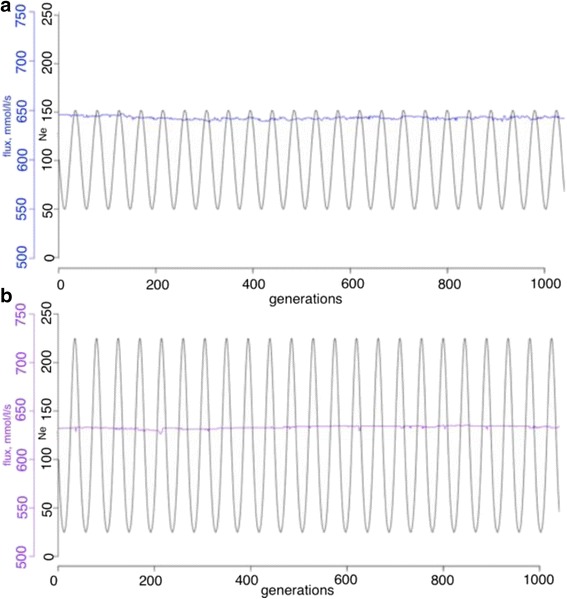
Fluxes overlaid with the experimental design are shown for fluctuating experiments with an explicit population and a fluctuating population size are shown. **a**. The flux (*blue line*) and the experimental design (*black line*) for experiment N2 are shown. **b**. The flux (*purple line*) and the experimental design (*black line*) for experiment N5 are shown

**Fig. 7:**
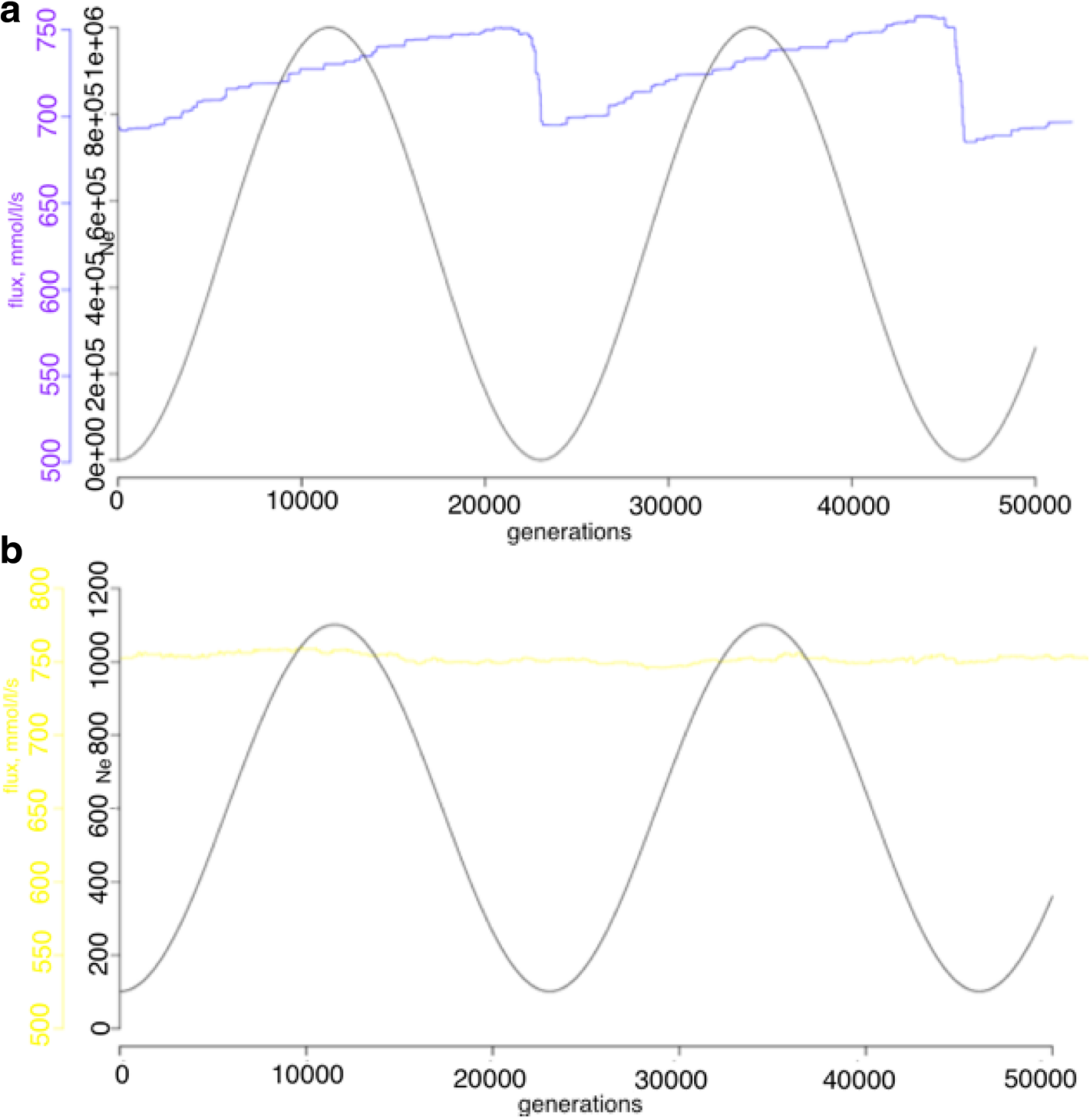
Fluxes overlaid with the experimental design are shown for fluctuating experiments with an explicit population and a fluctuating population size are shown. **a**. The flux (*blue line*) and the experimental design (*black line*) for experiment K4 are shown. **b**. The flux (*yellow line*) and the experimental design (*black line*) for experiment N5 are shown

Overall, elevated evolutionary stability of rate-limiting steps could be found in a limited range of parameters space (both of amplitude and periodicity). As can be seen from the Figs. 6 and 7, different range of the amplitude and periodicity cause different system responses. An extremely large amplitude (Fig. 7a) brings an adaptive response of the system fitness and decreased evolutionary stability of rate-limiting steps (Fig. 4), while less extreme schemes of parameter change (Figs. 6b and 7b) generate stable fitness with no directional changes and elevated evolutionary stability of rate-limiting steps. Some schemes (Fig. 6a) show no directional changes but fail to show elevated evolutionary stability of rate-limiting steps, as this appears to be tuned to the mutation rate and effect size of the population with the amplitude and frequency of the differences that would enable a response to occur. Overall, this does not support a hypothesized role for non-equilibrium processes in generating different rates of equilibration at different steps and corresponding different flux control during the adaptation process, at least in the conditions tested here.

### Experiments with an explicit population and a fluctuating asymptotic flux

**Fig. 8:**
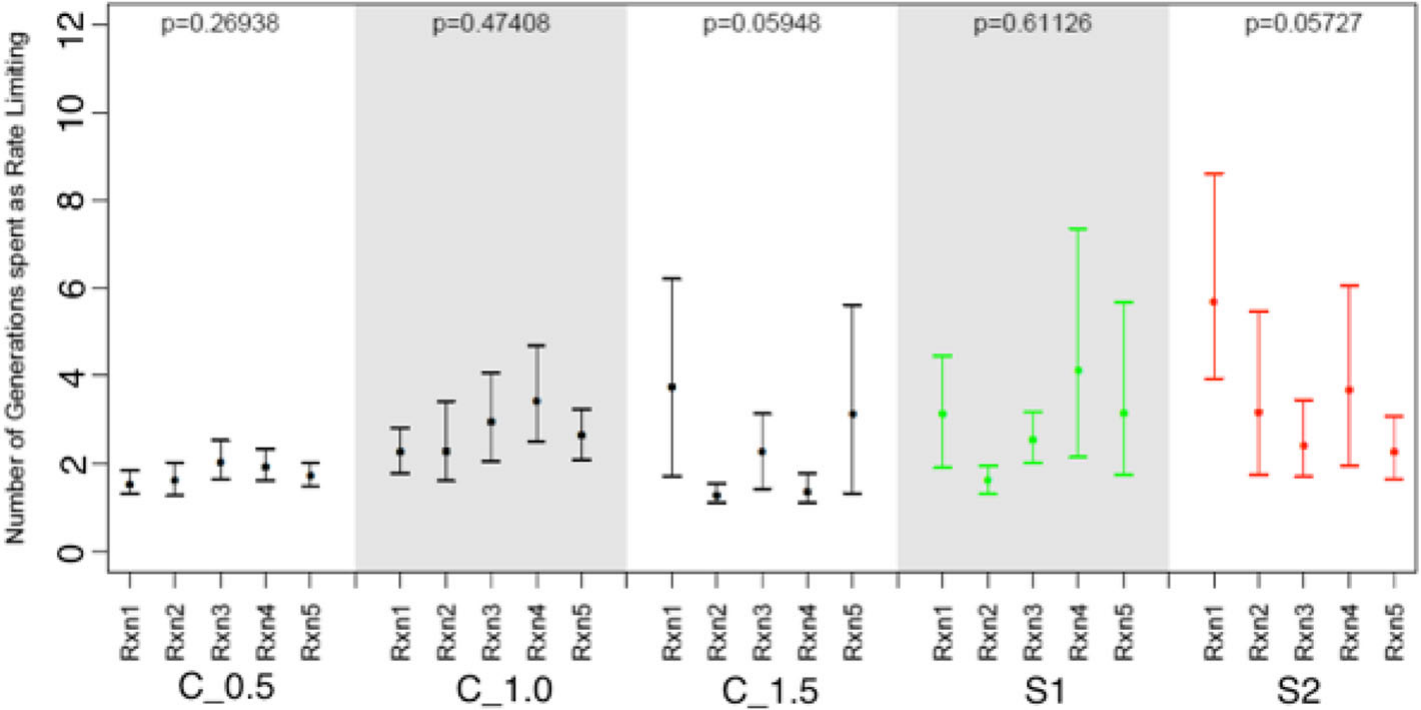
The distributions of the average lengths of rate-limiting steps between the reactions in the fluctuating selection experiments with an explicit population and fluctuating asymptotic flux, S1 (*green*), S2 (*red*), are shown. *Black bars* correspond to the control experiments with *a* = 0.5; (C_0.5), *a* = 1.00 (C_1.0), and *a* = 1.5 (C_1.5) correspondingly

**Fig. 9:**
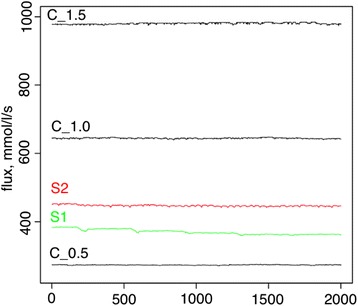
The fluxes of the experiments with an explicit population and fluctuating asymptotic flux, S1 (*green*), S2 (*red*), are shown. *Black lines* correspond to the control experiments with *a* = 0.5 (C_0.5), *a* = 1.00 (C_1.0), and *a* = 1.5 (C_1.5) correspondingly

**Fig. 10:**
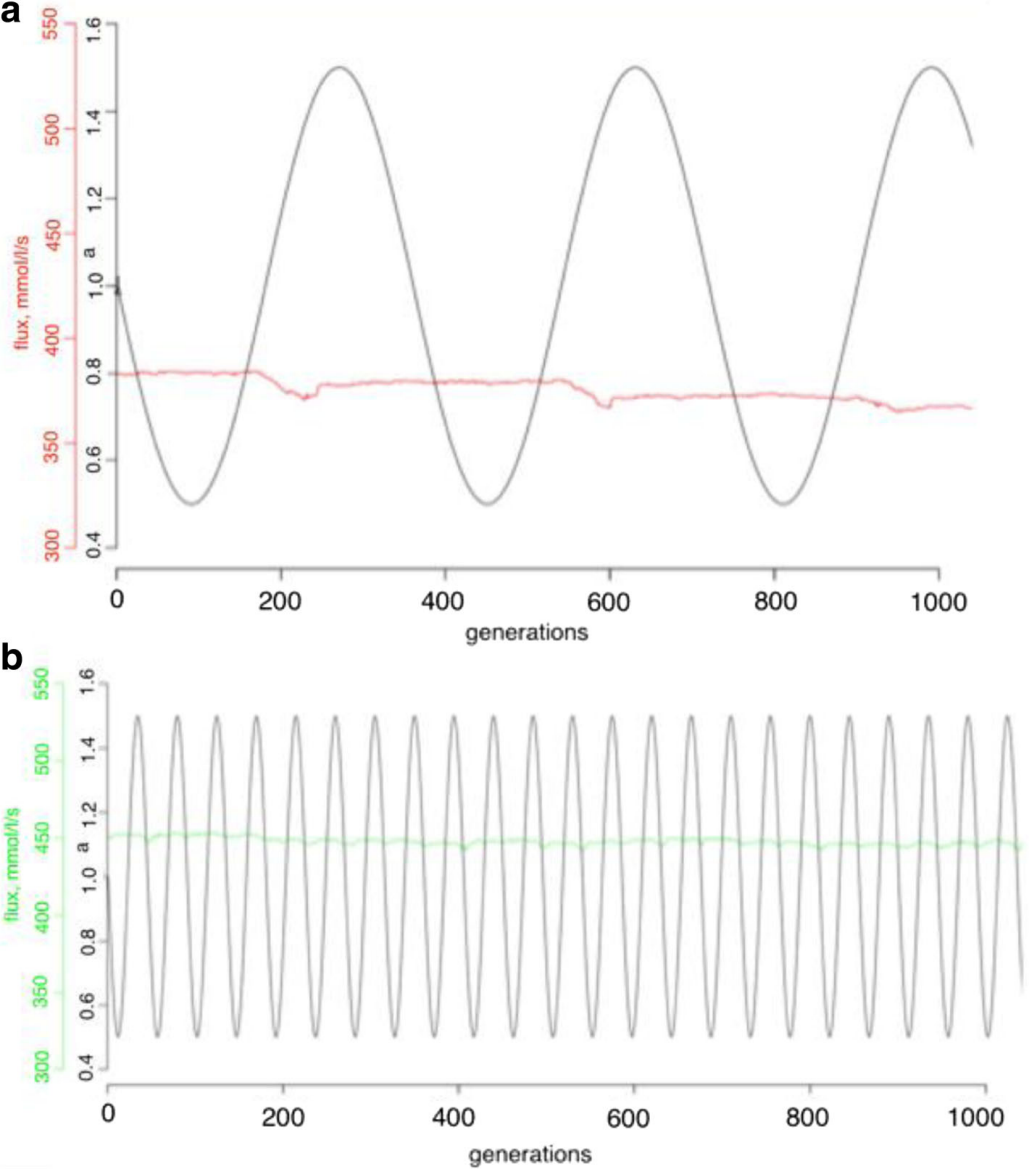
Fluxes and experimental designs of experiments with an explicit population and a fluctuating asymptotic flux are shown. **a**. The flux (*red line*) and the experimental design (*black line*) for experiment S1 are shown. **b**. The flux (*green line*) and the experimental design (*black line*) for experiment S2 are shown

Along with a fluctuating population size, fluctuating optimal flux was suggested to make a difference in evolutionary stability of rate-limiting steps, as this also has the potential to lead to non-equilibrium dynamics. Fluctuating selection in metabolic networks was studied previously and an increased robustness to changes was reported as a result of this fluctuating selection scheme [21]. Several schemes of fluctuating asymptotic flux were tested here, but only two were able to establish mutation-selection-drift balance because of numerical instability in solving the set of differential equations. (S1 and S2 (Additional file 1: Figure S4)). As can be seen in Fig. 8, there is no significant signal for elevated rate-limiting step stability in either experiment, although a slight increase in reaction stability in S2 was detected. Controls for the experimental schemes were assessed correspondingly at the highest, lowest, and middle point of schemes with *a* set to 0.5, 1.0 and 1.5. Fitness behavior over evolutionary time resembles adaptive changes for both schemes studied here (Additional file 1: Figure S4). A closer look at the combined plot of the amplitude and flux (Figs. 9 and 10) revealed that in both experiments, adaptive directional changes in flux correspond to fluctuations of asymptotic flux coefficients (a), as with population size. These observations support the findings above about the absence of elevated evolutionary stability of rate-limiting steps when fitness directional changes are present.

### Positive directional selection in an explicit population

**Fig. 11:**
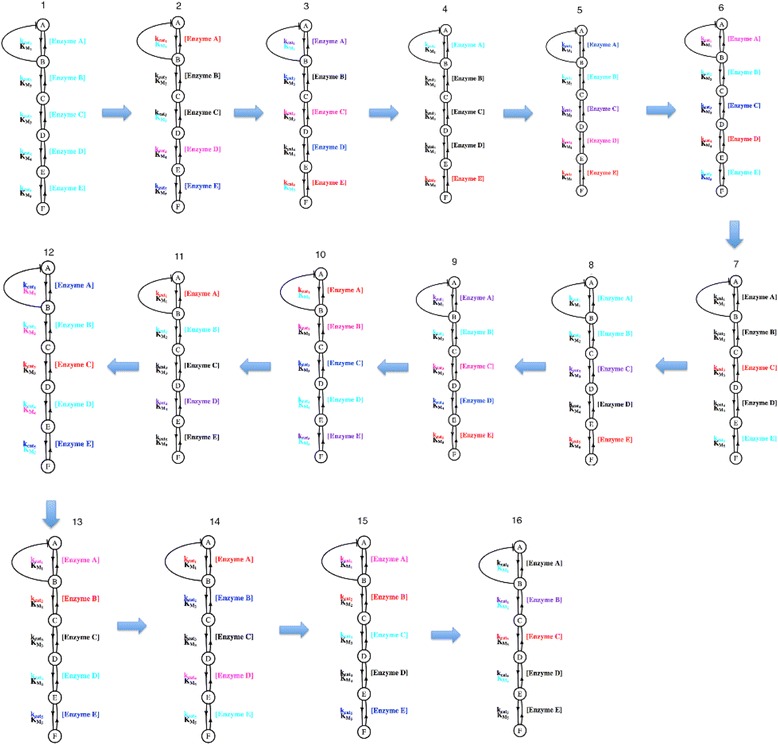
Clusters for significant co-evolving parameters during each evolutionary time regime were generated. This cluster analysis includes early and late periods of equilibrium surrounding a longer period of adaptation. Parameters that show the same color belong to the same cluster and co-evolve together, while *black* parameters are not significantly part of any cluster

To complement evolution with negative selection, a scheme involving positive selection was introduced. Selective pressures here directionally changed the flux. The co-evolution of enzyme activities across the pathway during adaptation is poorly understood, but presents a major challenge in generating a basic understanding of adaptive processes and differentiation from compensatory processes reflecting stabilizing selection. Co-evolutionary analysis was performed for the positive selection experiment with an explicit population. Cluster analysis estimated co-evolutionary relationships between various enzymatic parameters at different stages, the pre-adaptation equilibrium state (stage 1), during adaptation (stage 2–8), and late adaptation towards equilibrium (stage 9 on) (Fig. 1). As was expected, there are differences between the described stages (Fig. 11). Both equilibrium stages (1 and 16) contain clustered enzymatic parameters that belong to the same enzyme (Enzyme A and Enzyme C for stage 1 and Enzymes B and C for stage 16), similar to patterns observed for negative selection on flux only in previous studies [5]. The stages not in equilibrium showed different distributions of clusters. Stage 2 has the first 2000 generation after adaptive shift and is similar to stage 1 by containing intra enzyme clustered parameters. Stages 3–8 all contain various combination of clusters with inter- and intra-enzyme parameters clustered together, but never just intra-enzymes parameters clustered alone.

**Fig. 12:**
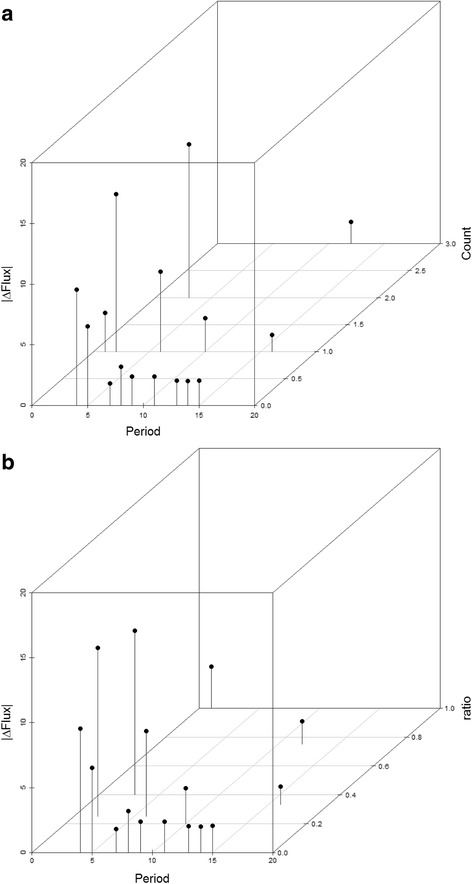
The association between the change in flux and the time period (and associated evolutionary regime) are compared with the count of inter-molecular pairwise parameter clusters (**a**) and the ratio of inter-molecular to intra-molecular pairwise clusters (**b**) per period

Since mutations that required increasing flux values come from the beneficial part of the mutational distribution, this process became time consuming, in taking 28,000 generations to reach equilibrium. The rate of adaptation varies at the different stages. The earlier and middle stages (2–5) show more rapid accumulation of beneficial changes and are responsible for most of the new equilibrium flux value gain, as might be expected. Mixed-effects regression analyses were performed in order to assess the association between flux change and the ratio/count of clusters across stages of adaptation, shown Fig. 12.

Mixed-effects regression analyses were performed in order to assess the association between flux change and the ratio/count of clusters across stages of adaptation, summarized numerically in Table 3 and graphically in Fig. 12. A statistically significant association was found between the count of inter-molecular clusters and the change in flux at the 0.05 level (*p* = 0.0424). However, a corresponding association was not found between the ratio of inter-molecular clusters and the change in flux at the 0.05 level (*p* = 0.3264). Overall, this provides some suggestive evidence that high changes in flux may have an effect on the amount of inter-molecular coevolution occurring at any given time and can be informative in analyzing the sequence co-evolution of enzymes in metabolic pathways from comparative genomic analysis. The exact patterns will be indicative of features of the enzymes and the fitness landscape.

**Table 3:** A statistical analysis is presented to evaluate the role of adaptation in driving inter-molecular vs. intra-molecular patterns of co-evolution

	Slope estimate	Standard error	*p*-value
Count	0.04227	0.0237	0.0424
Ratio	0.00326	0.00811	0.3264

Clusters that reflect co-evolving parameters were evaluated in cluster count and in the ratio of the inter- to intra-molecular clusters observed over different periods in Fig. 1. This was done, both during periods of adaptation and with an observation of a lag period for a response. Slope estimates are the main effect from the mixed-effects regression analyses, and standard errors are estimated based on the null distribution of slopes generated by the permutation test. The *p*-values were obtained by counting the proportion of the generated null distribution that was greater than the slope estimates

## Discussion

Here, various demographic and selective scenarios were tested in order to find out if specific population and selection parameters when systems may be out of equilibrium can give rise to a more evolutionary stable rate-limiting steps, following from previous work [5]. Further, we sought to examine if there was a systematic difference in the patterns of co-evolution between positive diversifying selection and compensatory covariation that might be predictive. Our findings here suggest that there is a specific range of population size fluctuations that cause elevated evolutionary stability of rate-limiting steps. It can be seen from two experimental frameworks, with an explicit population where scheme N5 generated the highest stabilities, while a decrease in periodicity representing more extreme shifts in population size don’t lead to an increase in the evolutionary stability of rate-limiting steps, but instead slightly decrease it (N6). Increased amplitude was shown to have a major impact in increasing rate-limiting steps stability. To investigate further the influence of the amplitude in fluctuating population size experiments, a switch to a non-explicit population experimental scheme was necessitated as the explicit scheme become too computationally intensive.

A number of schemes were implemented in the experiments with a calculated mutational fixation probability (Additional file 1: Figure S3). Surprisingly, assigning the amplitude to a higher range [100; 1,000,000] and maintaining the periodicity that was used in the previous experiments (explicit N2) didn’t give an increased rate-limiting step stability. Instead, the average consecutive length was slightly smaller than controls. It was possible that the values of periodicity and amplitude were too extreme and more relaxed amplitude and periodicity values were tested. Experimental scheme K4 showed the highest signal of stability from our sampling, suggesting that a reduced amplitude has a major impact on rate-limiting step evolutionary stability and that significant stability increase potentially exists in a certain periodicity and amplitude range, which would need to be established further by parameter sampling.

Positive diversifying selection resulted in an increase in the co-evolution of parameters that belong to different enzymes when compared with compensatory covariation under negative selection. In previous work [5], it was observed that more complex stabilizing selective or mutational schemes could also give rise to increases in inter-molecular parameter co-evolution, so the null model of stabilizing selection needs to be properly tuned to use this result in the identification of positive directional selection. The particular results observed in this study and the nature of the ridge in the fitness landscape are dependent upon the structure and the constraints of the pathway, which is treated in isolation here. While in an actual cell pathways may be less isolated and this is likely to change the size and nature of the fitness ridge of optima, we expect that the conclusions of this and prior work upon which this builds [5] are generalizable.

It had been hypothesized that constant adaptation when out of equilibrium would lead to different rates of adaptation in different parts of the pathway and the emergence of flux control, but this was not what was observed. This observation relates to patterns of evolution in changing environments that correspond to generalists vs. specialists. Specialist species are affected by isolation of their populations; they follow scenarios where there is constant rapid local adaptation. Specialists are known for stronger genetic differentiation among populations due to small and sometimes fluctuating population sizes with high frequencies of genetic bottlenecks [22]. Scenarios where this constant rapid adaptation does not happen are found for more generalist species. Generalists often have high genetic diversities in their populations and low genetic differentiation among them [23, 24]. This is the consequence of the absence of genetic bottlenecks and strong gene flow among populations. In regards to the overall evolutionary rate it is thought that specialists adapt faster than generalists to any given set of environmental conditions [25]. Several studies have reported rates of adaptation that are consistent with the prediction that specialists evolve faster than generalists [26, 27]. Long-term environmental variation may not lead to the evolution of generalists in all population genetic scenarios. Instead repeated evolution of specialists adapted to each set of growth conditions happens, as has been observed in adaptation of bacteriophage to alternate hosts [28].

## Conclusion

This study discovers that demographic and ecological variation has a direct impact on the evolutionary dynamics of metabolic pathways. Population size fluctuations when following a particular scheme with tuned periodicity facilitates increasing the evolutionary stability of flux-control points in metabolic pathway. Adaptation itself is a factor that changes co-evolutionary dynamics and adds a particular signal on top of the corresponding dynamics associated with stabilizing selection and compensatory processes. While at the mutation-selection-drift balance in a simple selective scheme, compensatory intra-enzyme mechanisms dominate, while during adaptive directional processes, inter-molecular parameters within the system co-evolve with stronger signals. Overall, these studies give a picture of the nature of pathway evolution under more complex selective and population genetic schemes and gives the potential to develop methods that might detect such scenarios from comparative sequence evolution patterns.

## Additional files

Additional file 1: Table S1. This table shows the ratio of positive fitness change counts, negative fitness change counts, total positive fitness change, total negative fitness change and total fitness change per evolutionary simulation step. **Table S2.** This table shows the average fitness for the first 1000 generations of each simulation step, the average fitness for the second 1000 generations of simulation step and *p*-values of Mann-Whitney’s test comparing fitness values of the first and the second halves of the simulation step. **Figure S1.** The simplified pathway that was simulated is shown. This pathway contains features from glycolysis [26]. A constant concentration of compound A is converted to compound F and the steady state flux is measured. **Figure S2.** Schemes of the experiments with an explicit population and a fluctuating population size are shown. The schemes for experiments N1 (green), N2 (blue), N3 (yellow), N4 (red), N5 (purple), N6 (brown) are shown. Black lines correspond to the control experiments with population sizes 25, 50, 100, 150, and 225. **Figure S3.** Schemes of the experiments with a calculated fixation probability and with fluctuating population size. A. The schemes for experiments K1 (green), K2 (red), K3 (blue) are shown. B. The schemes for the experiments K3 (blue), K4 (yellow), K5 (purple) are shown. Black lines correspond to the control experiments with population size 100, 1000, 1,000,000. **Figure S4.** Schemes of the experiments with an explicit population and fluctuating asymptotic flux. S1 (green) and S2 (yellow). Black lines correspond to the control experiments with *a* set to 0.5, 1.0, 1.5, corresponding to flux amplitudes of 325, 650, and 975. (DOCX 9986 kb)

## Abbreviations

[enzyme]: This is the concentration of the enzyme
k_cat_: This is the catalytic constant for the enzyme
k_catr_: This is the catalytic constant of the reverse reaction for the enzyme
K_m_: This is the Michaelis constant for the enzyme
K_mr_: This is the Michaelis constant of the reverse reaction for the enzyme
