# Supplementary material for: Characterizing the roles of changing population size and selection on the evolution of flux control in metabolic pathways
Source: BMC Evol Biol. 2017 May 25;17:117. doi: 10.1186/s12862-017-0962-7 (PMC5445498; doi:10.1186/s12862-017-0962-7)

Supplemental Materials

Characterizing the roles of changing population size and selection on the evolution of flux control in metabolic pathways

Alena Orlenko^1,2^, Peter B. Chi^1,3^, and David A. Liberles^1,2,^*

1. Department of Biology and Center for Computational Genetics and Genomics, Temple University, Philadelphia, PA 19122, USA
2. Department of Molecular Biology, University of Wyoming, Laramie, WY 82071, USA
3. Department of Mathematics and Computer Science, Ursinus College, Collegeville, PA 19426, USA

*address correspondence to DAL at daliberles@temple.edu; Other authors: AO: aorlenko@uwyo.edu; PBC: pchi@ursinus.edu

Table S1. This table shows the ratio of positive fitness change counts, negative fitness change counts, total positive fitness change, total negative fitness change and total fitness change per evolutionary simulation step.

| Evolutionary simulation step | Ratio of positive ΔFitness counts | Ratio of negative ΔFitness counts | Total positive ΔFitness | Total negative ΔFitness | Total ΔFitness |
| --- | --- | --- | --- | --- | --- |
| Step 1 | 0.021 | 0.979 | 0.13 | -36.98 | -36.85 |
| Step 2 | 0.9995 | 0.0005 | 24.51 | 0.0 | 24.51 |
| Step 3 | 0.9885 | 0.0115 | 54.99 | -0.01 | 54.98 |
| Step 4 | 0.9805 | 0.0195 | 82.24 | -0.05 | 82.19 |
| Step 5 | 0.9945 | 0.0055 | 79.09 | -0.02 | 79.07 |
| Step 6 | 0.965 | 0.035 | 100.57 | -0.20 | 100.37 |
| Step 7 | 0.8855 | 0.1145 | 16.94 | -0.7 | 16.24 |
| Step 8 | 0.994 | 0.006 | 69.01 | -0.01 | 68.99 |
| Step 9 | 0.9595 | 0.0405 | 39.37 | -0.20 | 39.17 |
| Step10 | 0.913 | 0.087 | 25.18 | -1.13 | 24.05 |
| Step 11 | 0.704 | 0.296 | 19.28 | -3.77 | 15.51 |
| Step 12 | 0.9965 | 0.0035 | 50.89 | -0.03 | 50.87 |
| Step 13 | 0.68 | 0.32 | 17.80 | -4.81 | 12.99 |
| Step 14 | 0.9105 | 0.0895 | 24.96 | -0.93 | 24.03 |
| Step 15 | 0.768 | 0.232 | 17.02 | -3.20 | 13.82 |
| Step 16 | 0.001 | 0.999 | 0.00 | -63.55 | -63.55 |

Table S2. This table shows the average fitness for the first 1000 generations of each simulation step, the average fitness for the second 1000 generations of simulation step and p-values of Mann-Whitney’s test comparing fitness values of the first and the second halves of the simulation step.

| Evolutionary simulation step | Average fitness for the first half of generations in a given step | Average fitness for the second half of generations in a given step | p-value |
| --- | --- | --- | --- |
| Step 1 | 0.4 | 0.4 | 0.15 |
| Step 2 | 0.03 | 0.04 | 0.0 |
| Step 3 | 0.06 | 0.09 | 0.0 |
| Step 4 | 0.12 | 0.17 | 0.0 |
| Step 5 | 0.2 | 0.24 | 0.0 |
| Step 6 | 0.29 | 0.33 | 0.0 |
| Step 7 | 0.35 | 0.36 | 3.94e-239 |
| Step 8 | 0.39 | 0.41 | 2.44e-272 |
| Step 9 | 0.43 | 0.45 | 7.43e-286 |
| Step10 | 0.47 | 0.47 | 7.15e-62 |
| Step 11 | 0.48 | 0.5 | 5.52e-244 |
| Step 12 | 0.52 | 0.52 | 7.37e-48 |
| Step 13 | 0.52 | 0.54 | 8.64e-241 |
| Step 14 | 0.57 | 0.57 | 0.0016 |
| Step 15 | 0.58 | 0.58 | 2.39e-26 |
| Step 16 | 0.57 | 0.55 | 8.42e-202 |

Figure S1. The simplified pathway that was simulated is shown. This pathway contains features from glycolysis [25]. A constant concentration of compound A is converted to compound F and the steady state flux is measured.


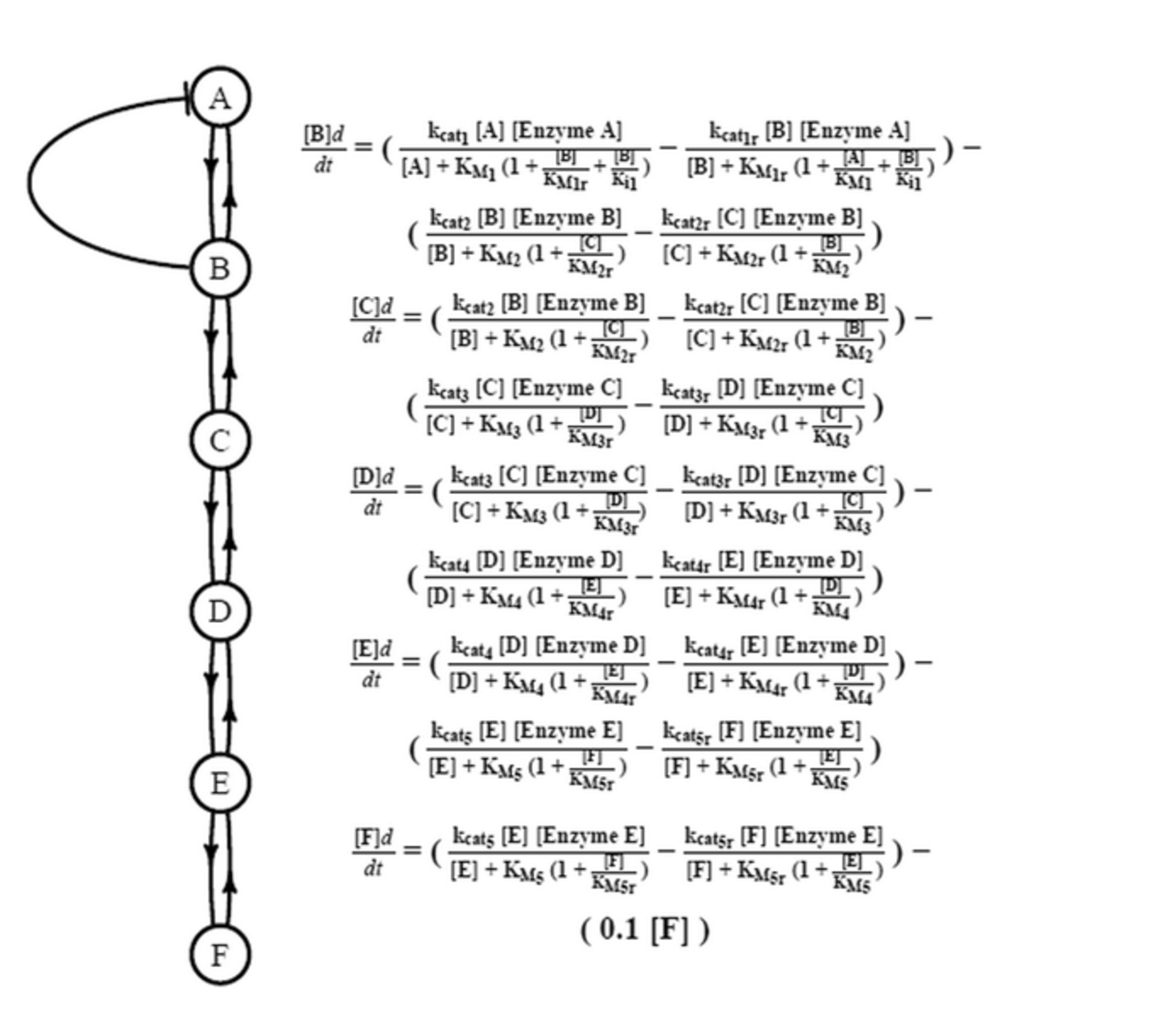


Figure S2. Schemes of the experiments with an explicit population and a fluctuating population size are shown. Shown the schemes of experiments N1 (green), N2 (blue), N3 (yellow), N4 (red), N5 (purple), N6 (brown). Black lines correspond to the control experiments with population size 25, 50, 100, 150, 225.


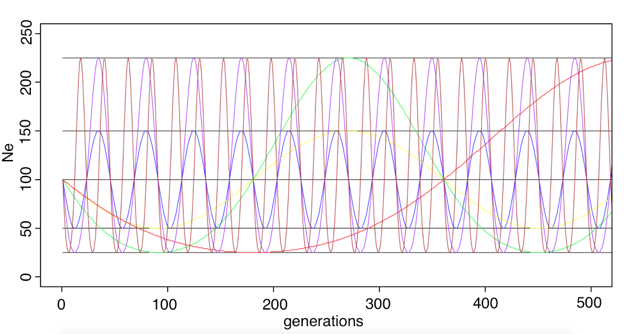


Figure S3. Schemes of the experiments with a calculated fixation probability and fluctuating population size. A. Shows the schemes of experiments K1 (green), K2 (red), K3 (blue). B. shows the schemes of the experiments K3 (blue), K4 (yellow), K5 (purple). Black lines correspond to the control experiments with population size 100, 1000, 1000000.


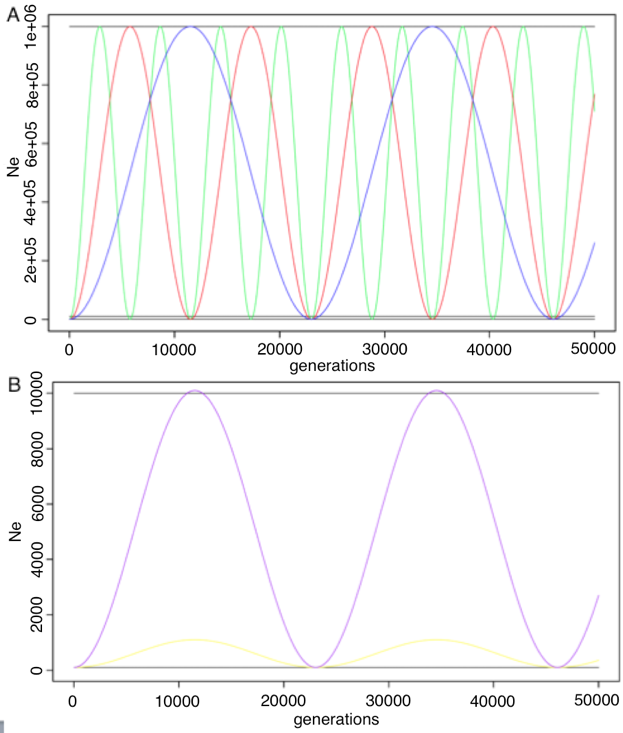


Figure S4. Schemes of the experiments with an explicit population and fluctuating asymptotic flux. S1 (green) and S2 (yellow). Black lines correspond to the control experiments with *a* set to 0.5, 1.0, 1.5, corresponding to flux amplitudes of 325, 650, and 975.


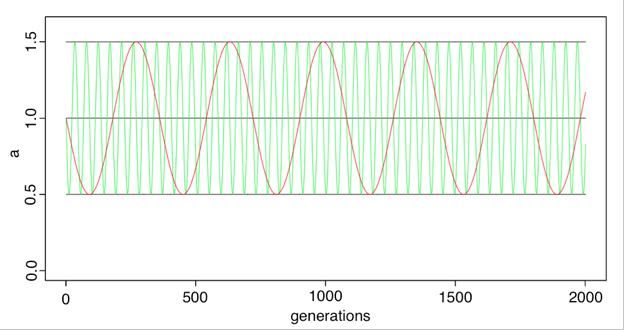

Supplement: Supplementary file 1 — This table shows the ratio of positive fitness change counts, negative fitness change counts, total positive fitness change, total negative fitness change and total fitness change per evolutionary simulation step. Table S2. This table shows the average fitness for the first 1000 generations of each simulation step, the average fitness for the second 1000 generations of simulation step and p-values of Mann-Whitney’s test comparing fitness values of the first and the second halves of the simulation step. Figure S1. The simplified pathway that was simulated is shown. This pathway contains features from glycolysis [26]. A constant concentration of compound A is converted to compound F and the steady state flux is measured. Figure S2. Schemes of the experiments with an explicit population and a fluctuating population size are shown. The schemes for experiments N1 (green), N2 (blue), N3 (yellow), N4 (red), N5 (purple), N6 (brown) are shown. Black lines correspond to the control experiments with population sizes 25, 50, 100, 150, and 225. Figure S3. Schemes of the experiments with a calculated fixation probability and with fluctuating population size. A. The schemes for experiments K1 (green), K2 (red), K3 (blue) are shown. B. The schemes for the experiments K3 (blue), K4 (yellow), K5 (purple) are shown. Black lines correspond to the control experiments with population size 100, 1000, 1,000,000. Figure S4. Schemes of the experiments with an explicit population and fluctuating asymptotic flux. S1 (green) and S2 (yellow). Black lines correspond to the control experiments with a set to 0.5, 1.0, 1.5, corresponding to flux amplitudes of 325, 650, and 975. (DOCX 9986 kb) [file 12862_2017_962_MOESM1_ESM.docx]
